# Serum Chemerin Levels in Polish Women with PCOS-Phenotype D

**DOI:** 10.3390/jcm15020772

**Published:** 2026-01-17

**Authors:** Justyna Kuliczkowska-Płaksej, Jowita Halupczok-Żyła, Łukasz Gojny, Agnieszka Zembska, Aneta Zimoch, Monika Skrzypiec-Spring, Marek Bolanowski, Aleksandra Jawiarczyk-Przybyłowska

**Affiliations:** 1 Department of Endocrinology and Internal Diseases, Wroclaw Medical University, 50-367 Wrocław, Poland; lukasz.gojny@umw.edu.pl (Ł.G.); agnieszka.zembska@umw.edu.pl (A.Z.); aneta.zimoch@usk.wroc.pl (A.Z.); marek.bolanowski@umw.edu.pl (M.B.); 2 Department of Clinical and Experimental Patology, Wroclaw Medical University, 50-367 Wrocław, Poland; jowita.halupczok-zyla@umw.edu.pl; 3 Department of Pharmacology, Wroclaw Medical University, 50-367 Wrocław, Poland; monika.skrzypiec-spring@umw.edu.pl

**Keywords:** polycystic ovary syndrome, chemerin, low grade inflammation, adipokines, adipose tissue, fat, body mass index

## Abstract

**Objectives:** Polycystic ovary syndrome (PCOS) is a heterogeneous disorder with diverse pathogenetic mechanisms and clinical manifestations. Phenotype D PCOS is characterized by oligomenorrhoea and polycystic ovaries without hyperandrogenism. Altered adipokine profiles may contribute to reproductive and metabolic disturbances. Chemerin is an adipokine involved in inflammatory and metabolic processes. It remains unclear whether altered chemerin levels in PCOS reflect metabolic dysfunction alone or are directly associated with hyperandrogenism. The aim of this study was to compare serum chemerin levels in women with normoandrogenic PCOS and a control group. **Methods:** This cross-sectional preliminary study included 49 women with phenotype D PCOS and 40 healthy, age- and body mass index (BMI)-matched controls. Anthropometric, biochemical, hormonal parameters, and serum chemerin concentrations were assessed. **Results:** Serum chemerin concentrations did not differ significantly between the groups. In the PCOS group, the 95% confidence interval ranged from 198.61 to 234.37, while in the controls, it ranged from 187.13 to 216.21. In women with PCOS, chemerin showed significant positive correlations with weight, BMI, waist and hip circumference, total adipose tissue, and both gynoid and android fat content. Positive correlations were also observed with highly sensitive C-reactive protein (hs-CRP), insulin, glucose, triglycerides, and Homeostasis Model Assessment of Insulin Resistance (HOMA-IR), and a negative correlation was found with high-density lipoprotein (HDL) cholesterol. Chemerin was weakly negatively correlated with sex hormone binding globulin (SHBG) and positively correlated with the free androgen index (FAI). In the control group, chemerin correlated positively with CRP, insulin, triglycerides, total and gynoid adipose tissue, and negatively correlated with HDL cholesterol and SHBG. **Conclusions** Although chemerin levels did not differ from controls, chemerin was associated with metabolic and inflammatory markers in both groups. These findings should be considered preliminary due to the limited sample size. Chemerin may reflect metabolic and inflammatory status rather than hyperandrogenism in normoandrogenic PCOS.

## 1. Introduction

Polycystic ovary syndrome (PCOS) is one of the most prevalent endocrine disorders in women of reproductive age, affecting an estimated 5–25% of this population [1]. Beyond menstrual and fertility disturbances, the condition is linked to an increased risk of cardiovascular and metabolic complications [2,3,4,5]. Both the pathogenesis of PCOS and its clinical presentation are highly heterogeneous [2,6,7]. The rare normoandrogenic phenotype D remains particularly controversial, largely due to its distinct clinical features. The etiology of PCOS is believed to result from a combination of genetic, epigenetic, metabolic, endocrine, and immune influences [2,6,7].

Adipose tissue—the body’s largest endocrine organ—secretes numerous bioactive molecules, including adipokines. In reproductive biology, both clinical and experimental studies have shown that certain adipokines play important roles in regulating reproductive function and pregnancy. Altered adipokine profiles, particularly in the context of overweight and obesity, may contribute to reproductive disorders [8]. Chemerin is an adipokine that has gained increasing attention in this context. Human studies demonstrate positive correlations between circulating chemerin levels and body mass index (BMI), triglycerides, C-reactive protein, and blood pressure [9,10,11]. Some findings also indicate that chemerin may impair FSH-stimulated steroidogenesis in ovarian follicular cells [6,8,12,13,14]. Growing evidence suggests that the chemerin signaling system participates in normal reproductive processes and may be involved in the pathophysiology of several reproductive disorders, including PCOS [15,16,17,18]. However, its exact role remains incompletely understood, particularly within reproductive biology and gynecology. Chemerin appears to be linked to multiple mechanisms implicated in PCOS pathogenesis, such as obesity, hyperinsulinemia, low-grade inflammation, and impaired ovarian steroidogenesis [19,20,21,22]. It may contribute to several hallmark features of PCOS, including ovarian dysfunction, hyperandrogenism, and adverse reproductive outcomes, while also showing associations with the metabolic abnormalities common in PCOS [19,20,21,22]. Nevertheless, the degree to which chemerin contributes to PCOS and its metabolic complications remains inconsistent across studies.

The aim of our study was to evaluate chemerin concentrations in women with phenotype D PCOS and compare these values to those of age-, weight-, and BMI-matched healthy controls.

## 2. Materials and Methods

**Figure 1:**
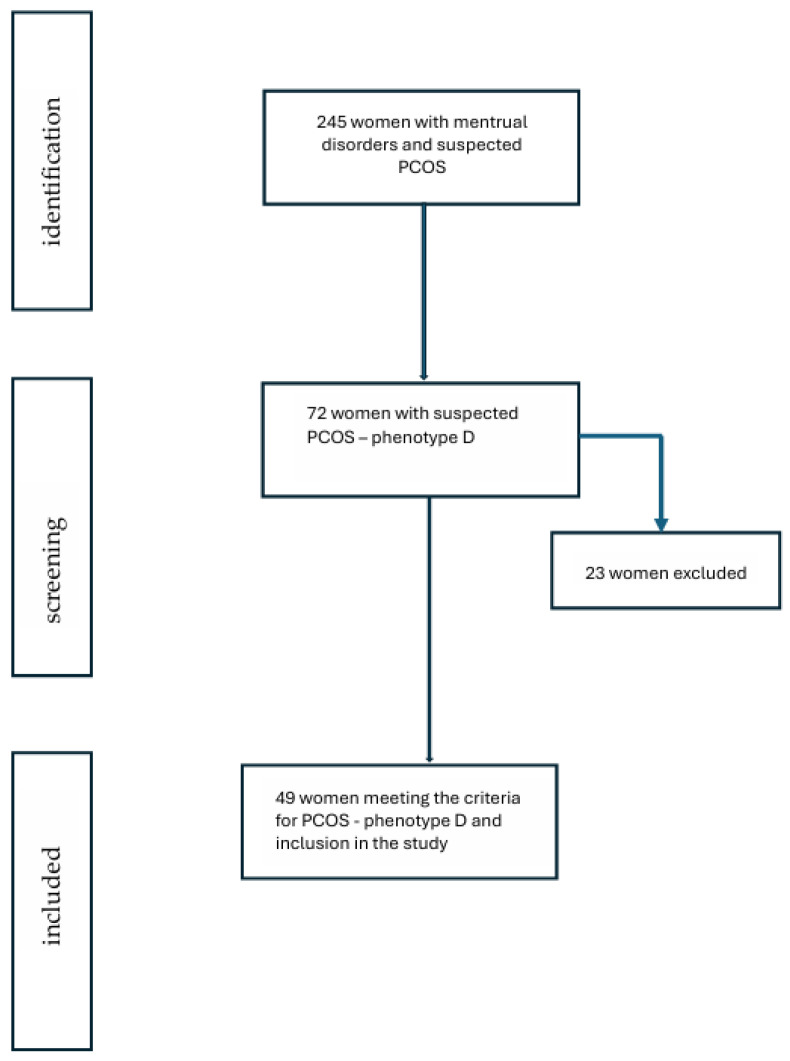
Flow chart of the patients included in the analysis.

This is a cross-sectional preliminary study. The study was performed on an ethnically homogenous population of 89 women aged 27–38 years, from the Lower Silesia Region in Poland, including 49 women with PCOS and 40 healthy, age-, weight-, and BMI-matched controls. All PCOS patients were selected from the Department of Endocrinology and Internal Diseases, University Hospital in Wroclaw, between January 2024 and December 2025. (Figure 1).

All women in the PCOS group presented with phenotype D, i.e., oligomenorrhoea and polycystic ovaries on ultrasound without biochemical and/or clinical hyperandrogenism, according to Rotterdam’s criteria [2]. The control group was recruited from healthy volunteers with regular and ovulatory menstrual cycles.

Exclusion criteria were pregnancy, drug use (such as glucocorticoids, oral contraceptives, statins, fibrates, and metformin), alcohol and tobacco use, chronic, inflammatory, or neoplastic disorders, cardiovascular disorders, hypertension, diabetes, hypo- and hyperthyroidism, hypercortisolemia, 21-hydroxylase deficiency, hyperprolactinemia, and virilizing adrenal or ovarian tumors. Patients had not participated in a special diet. Patients who had previously been treated with anti-obesity medications, as well as those who had undergone bariatric surgery, were also excluded.

Anthropometric measurements were performed for all patients according to the World Health Organization: weight and height and waist and hip circumference.

BMI and waist-to-hip ratio (WHR) were calculated using the standard formulas: BMI = body mass (kg)/height (m)^2^, WHR = waist circumference (cm)/hip circumference (cm).

The Homeostatic Model Assessment—Insulin Resistance (HOMA-IR) was calculated from the following formula: [fasting glucose [nmol/L] × (fasting insulin [mUI/L])/22.5]. The Free Androgen Index (FAI) was calculated using the formula testosterone [nmol/L] × 100/sex hormone binding globulin (SHBG) [nmol/L] according to Vermeulen [23].

Blood samples were drawn from the cubic vein in the morning after an overnight fast at the follicular phase (between 3rd and 6th day of menstrual cycle), and immediately centrifuged at +4° C and stored at −20 °C.

The serum levels of chemerin were measured by a commercial sandwich enzyme immunoassay (RD191136200R Human Chemerin ELISA, BioVendor, Brno, Czech Republic). The intra- and inter-assay coefficients of variation were 5.1–7% and 6.9–8.3%, respectively.

Biochemical and hormonal parameters included glucose, total cholesterol (TC), high-density lipoprotein cholesterol (HDL-C), triglycerides (TG), low-density lipoprotein cholesterol (LDL-C), high-sensitive C-reactive protein (hs-CRP) (measured using the enzymatic method at Alinity by Abbott Laboratories, Chicago, IL, USA); insulin, luteinizing hormone (LH), follicle-stimulating hormone (FSH), testosterone and androstenedione, and sex hormone-binding globulin (SHBG) (measured using the chemiluminescent method from Immulite 2000 XPi by Siemens Healthineers, Forchheim, Germany).

The percentage of total adipose tissue, gynoid, and android depots of adipose tissue was assessed using the dual-energy X-ray absorptiometry (DXA) method (Horizon A Hologic densitometer, Hologic, Inc., Marlborough, MA, USA).

Ovarian ultrasound was performed on all participants between the 6th and 10th days of the menstrual cycle. The Ferriman–Gallwey score (to evaluate and quantify hirsutism) was used in all participants.

The bioethics committee of Wroclaw Medical University approved the protocol of the study (approval decision number: KB192/2023N). All subjects signed informed consent forms in line with the Declaration of Helsinki.

### Statistical Analysis

**Figure 2:**
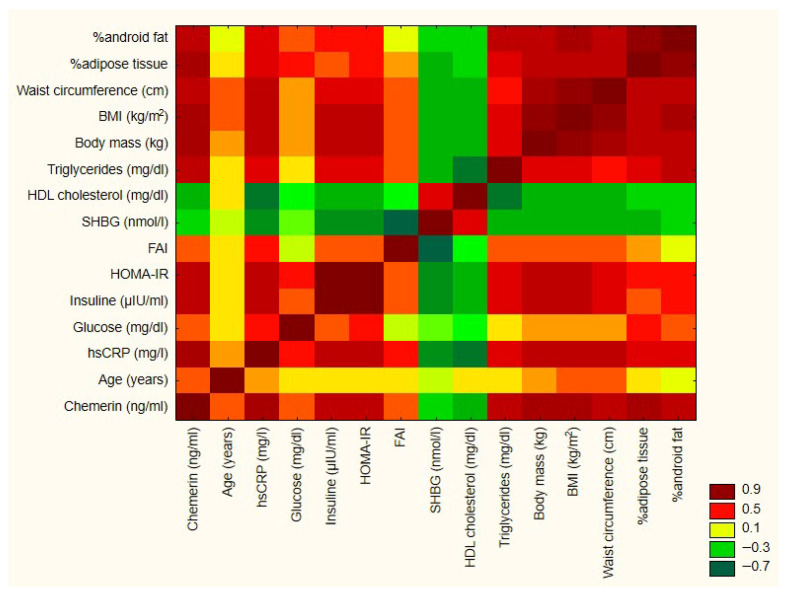
A heatmap of the correlation between variables.

Data were analyzed using Statistica software for Windows (version 13.3, StatSoft, Krakow, Poland). Results are presented as the mean with standard deviation (SD), median, and interquartile range (IQR). The Shapiro–Wilk test was used to assess data distribution. Student’s t-test or Mann–Whitney test was applied to compare quantitative variables. The homogeneity of variances was verified using Levene’s test. Correlations between parameters were calculated using Pearson’s test or Spearman’s rank correlation test. A *p*-value less than 0.05 was considered statistically significant. We used a heatmap to visualize correlations between variables (Figure 2).

## 3. Results

There were no statistically significant differences in weight or BMI between groups. Waist circumferences as well as hip circumferences were significantly higher in patients with PCOS than in the control group, although there was no difference in WHR value between the groups. We did not observe statistically significant differences in the content of adipose tissue (total, android, and gynoid) between the groups. The general characteristics of the study group and the control group are presented in Table 1.

In the PCOS group, we observed significantly higher testosterone, androstenedione, and LH concentrations as well as FAI values, although FAI values as well as androgen concentrations were generally within normal limits (Table 2). Hs-CRP was significantly higher in the PCOS group. The concentration of chemerin did not differ significantly between the studied groups. In the PCOS group, the 95% confidence interval ranged from 198.61 to 234.37, whereas in the control group, the 95% confidence interval ranged from 187.13 to 216.21.

In the PCOS group, chemerin was positively correlated with weight, BMI, waist and hip circumference, total adipose tissue, and gynoid and android adipose content (Table 3). We also observed a positive correlation with hs-CRP level, insulin, glucose, and triglyceride concentration and HOMA value, and a negative correlation with HDL-cholesterol level. SHBG was negatively correlated, and the FAI value was positively correlated with chemerin concentration (Table 3).

In the control group, chemerin concentration was negatively correlated with HDL-cholesterol and SHBG. We observed a positive correlation between chemerin concentration and C-reactive protein, insulin, triglycerides, gynoid adipose tissue content, and total adipose content in the control group (Table 4).

## 4. Discussion

PCOS is a heterogeneous condition with diverse etiologies and a wide spectrum of clinical presentations. International guidelines emphasize hyperandrogenism, ovulatory dysfunction, and polycystic ovarian morphology as the core features of the syndrome; however, the precise definition of PCOS remains a matter of debate [2,6,7]. Phenotype D of PCOS, also referred to as normoandrogenic PCOS, was introduced by the 2003 Rotterdam criteria [2]. In 2006, the Androgen Excess–PCOS Society (AE-PCOS) proposed excluding phenotype D from the diagnostic criteria, arguing that hyperandrogenism is essential for the diagnosis [24]. These criteria were never widely adopted, and the Rotterdam criteria remain the diagnostic standard. Several authors, including Unfer et al. [25], have argued for distinguishing hyperandrogenic phenotypes from phenotype D, suggesting that they may represent separate disorders with different etiologies [7,25,26,27]. Phenotype D is characterized by normal androgen levels, the mildest endocrine dysfunction, and the lowest prevalence of metabolic syndrome [27,28,29]. Consequently, patients with phenotype D do not exhibit clinical signs of hyperandrogenism, such as hirsutism, and typically present with a normal modified Ferriman–Gallwey score [27,30]. In contrast, phenotypes A, B, and C show an association between hyperandrogenism and metabolic disturbances [28,31]. Zhao et al. [32] demonstrated significantly higher HOMA-IR values in hyperandrogenic phenotypes compared with phenotype D, the HOMA-IR values of which were similar to those of the control group. Tripathy et al. [33] likewise reported the lowest frequency of metabolic syndrome in phenotype D.

In our study, we investigated women with phenotype D PCOS, the rarest PCOS phenotype in the Polish population [26]. The study and control groups were matched for age, weight, and BMI. As expected, androgen concentrations in phenotype D remained within the normal range, although they were still significantly higher than those in the control group. According to the Rotterdam criteria, when classifying patients as phenotype D, we considered testosterone levels and the FAI value. In some patients from this group, androstenedione levels slightly exceeded the upper limit of normal (which was 3.5 ng/mL), while testosterone and DHEA-S levels were within the normal range, and no clinical features of hyperandrogenism were present. Compared with healthy, regularly menstruating women of similar age and BMI and with normal ovarian morphology, phenotype D patients did not differ significantly in metabolic parameters despite their higher androgen levels. However, they did exhibit elevated hs-CRP levels, suggesting the presence of low-grade inflammation. Some studies have reported lower average BMI in phenotype D compared with hyperandrogenic phenotypes [34,35]. Higher levels of total cholesterol, LDL, and triglycerides have been observed in phenotype A relative to phenotype D, supporting the link between hyperandrogenism and metabolic comorbidities such as hyperinsulinemia and obesity. Dadachanji et al. [36] also found reduced levels of total cholesterol, LDL, triglycerides, and the ApoB:ApoA-1 ratio in phenotype D compared with phenotype A. The classification of phenotype D thus remains controversial, and some researchers consider it a separate condition.

Adipose tissue dysfunction, including disturbances in adipokine profiles, is one of the most widely studied alterations in PCOS [37].

Chemerin, an adipokine mainly expressed in white adipose tissue, the liver, and the placenta, but also in brown fat, lungs, kidneys, ovaries, skeletal muscle, and the heart [38,39], plays roles in inflammation, energy metabolism, adipogenesis, angiogenesis, and insulin secretion in adipose tissue and the ovaries [38].

It has been hypothesized that chemerin affects insulin signaling, potentially contributing to insulin resistance (IR) by disrupting the function of certain proteins in the insulin signaling cascade [40,41]. Many studies have revealed the role of chemerin in the development of obesity, IR, type 2 diabetes mellitus (T2DM), and gestational diabetes [42,43]. In most studies, chemerin levels were positively correlated with BMI, circulating lipids, blood pressure, and markers of poor glycemic control, fasting plasma glucose, fasting insulin, glycated hemoglobin (HbA1c), HOMA-IR, and different inflammation markers such as C-reactive protein, interleukin-6, and tumor necrosis factor α (TNF-α) [9,44,45]. Notably, these correlations remained consistent after adjusting for factors such as age, sex, and BMI [9,44,45]. High HOMA-IR, along with increased circulating chemerin levels, is characteristic of patients with metabolic syndrome [46,47]. Numerous studies indicate that chemerin levels are increased in obese adults and children [48,49]. Obese individuals (BMI > 30 kg/m^2^) have significantly higher chemerin levels than lean individuals, with levels decreasing after bariatric surgery [41]. Lifestyle modifications have also been shown to decrease chemerin levels and improve IR [50]. Furthermore, patients who have undergone bariatric surgery exhibit decreased circulating chemerin levels, emphasizing the strong correlation between chemerin concentration and adipose tissue mass [51,52]. However, some studies have yielded inconclusive results, showing both increased and unchanged chemerin levels in T2DM and obesity. Yilmaz et al. [53] did not observe a significant difference in serum chemerin levels between obese T2DM patients, non-obese T2DM patients, and the control group. One explanation may be that chemerin expression is influenced not only by adiposity but also by proteolytic processing, which can vary across metabolic states [54]. Such inconsistencies may also result from differences in sample size, age, lifestyle factors, and the presence of inflammatory factors. Moreover, previous reports have suggested that chemerin activity, rather than total concentration, may better reflect metabolic dysregulation in T2DM [53].

Several studies have demonstrated potential involvement of chemerin in ovarian physiology and reproductive outcomes [19,20]. Yang et al. [16] showed an association between chemerin and miscarriage in PCOS patients, while Huang et al. [55] reported correlations between chemerin and ovarian volume, follicle number, and SHBG levels. Chemerin concentrations were also significantly higher in follicular fluid and granulosa–lutein cells from PCOS patients with IR compared to those without IR [55]. In addition, a retrospective study confirmed the association between chemerin and ovarian polycystic changes in PCOS patients, and the results showed that serum chemerin concentration could reflect the severity of ovarian polycystic changes [21,55]. These studies suggest that chemerin might play a role in follicular dysplasia in PCOS patients, but the specific mechanism behind this phenomenon requires further investigation. Overall, studies on chemerin in PCOS remain scarce and yield inconsistent results [19,20].

In our study, we found no statistically significant difference in chemerin levels between women with phenotype D PCOS and matched controls. Within the PCOS group, chemerin levels showed strong positive correlations with hs-CRP, HOMA-IR, insulin concentrations, body weight, BMI, and body fat content. In the control group, chemerin also correlated—albeit weakly—with hs-CRP and metabolic indicators such as BMI, insulin, body fat content, and SHBG, although the correlations with BMI and SHBG were the weakest. These findings suggest that chemerin concentrations may be driven primarily by the severity of metabolic disturbances and the presence of obesity, especially in the case of PCOS.

The positive correlation between chemerin and hs-CRP further supports its relationship with inflammatory activity, which is consistent with numerous studies investigating chemerin’s roles in inflammatory cells. A positive correlation was observed between hs-CRP and chemerin concentration in experimental models of non-diabetic obese and non-diabetic non-obese patients. These markers were significantly higher in the non-diabetic obese group compared to the controls, further supporting chemerin’s pro-inflammatory role in obesity [56,57]. Chemerin receptors, including C-C motif chemokine receptor-like 2 (CCRL2) and chemokine-like receptor 1 (CMKLR1), have been identified on neutrophils, macrophages, mast cells, and in the ovaries [39,58,59,60]. Chronic low-grade inflammation has been recognized as one of the major causes and consequences of PCOS, as supported by alterations in inflammatory factors and cells in circulation as well as locally in the ovaries of PCOS patients [61,62,63]. In our study, women with PCOS demonstrated significantly higher hs-CRP levels compared to controls. Low-grade inflammation is a well-recognized feature of PCOS, particularly in classical phenotypes [63]. The degree of inflammation appears to be influenced by metabolic abnormalities as well as hyperandrogenemia [61,63,64]. It is, therefore, plausible that increasing hyperandrogenism and the simultaneous worsening of metabolic status may contribute to higher chemerin levels. In our cohort, the degree of inflammation in phenotype D was mild, which may reflect the unique nature of this phenotype.

A growing body of evidence indicates a link between chemerin and PCOS, and numerous studies have identified chemerin as an adipokine that may contribute to the pathophysiology of this syndrome [21]. Elevated circulating and ovarian chemerin levels were first demonstrated in animal models of PCOS induced by dihydrotestosterone, and more recent systematic reviews with meta-analyses have reinforced the strong association between serum chemerin concentrations and the diagnosis of PCOS [11]. Abruzesse et al. [65] reported higher chemerin levels in women with PCOS compared to controls, with no differences found between PCOS patients regardless of the presence or absence of metabolic syndrome, IR, or cardiovascular risk factors. These findings suggest that increased chemerin levels in PCOS occur independently of excess androgen or metabolic abnormalities [65]. Chemerin has also been shown to correlate with various metabolic parameters, although the specific associations differ across study populations. Some studies suggest a stronger association with components of metabolic syndrome, including obesity and IR, rather than with PCOS itself. Two meta-analyses demonstrated a significant relationship between PCOS and elevated chemerin levels that was independent of BMI, while also noting that chemerin tended to be higher in overweight and obese individuals [66,67]. Nevertheless, the exact influence of BMI and obesity on chemerin concentrations in women with PCOS remains uncertain. In the meta-analysis of 22 studies, chemerin levels were generally higher in the PCOS group compared to the non-PCOS group [66]. The subgroup analysis according to BMI revealed that serum chemerin levels were higher in women with PCOS and BMI > 25 kg/m^2^ compared to women from the non-PCOS group and BMI > 25 kg/m^2^. Women with PCOS and BMI < 25 kg/m^2^ did not show any significant increase in serum chemerin levels compared to the non-PCOS group with BMI < 25 kg/m^2^ [66]. Moreover, serum chemerin levels were significantly higher in women with PCOS and a higher BMI compared to women with PCOS and a lower BMI. Therefore, serum chemerin levels showed an independent relationship with BMI. In agreement with these findings, Guzel et al. [68] showed higher serum chemerin levels in women with PCOS and in women with both PCOS and obesity compared to women without PCOS and to those with PCOS and normal weight, respectively. In addition, they found higher chemerin levels in women with PCOS and obesity compared to women with PCOS and normal weight. Their results showed serum chemerin levels related to fat mass rather than to PCOS status. Increased serum chemerin levels were observed in women with PCOS and IR compared to women with IR but without PCOS. Moreover, serum and chemerin levels were higher in women with PCOS and IR compared to those with PCOS but without IR [68]. On the other hand, a case–control study with BMI- and weight-matched participants confirmed that chemerin levels were higher in women with PCOS than in controls, independent of adiposity [69]. In another cross-sectional study of 100 PCOS patients and 70 controls presenting with primary infertility, chemerin levels in the PCOS group showed significant positive correlations with fasting glucose, insulin, and HOMA-IR [18]. Similarly, Niepsuj et al. [70] found that serum chemerin in PCOS correlated positively with fasting insulin, fasting glucose, and HOMA-IR. Our findings are in line with reports suggesting that chemerin levels in normoandrogenic PCOS or in normal-weight PCOS patients do not significantly differ from those of age- and weight-matched healthy controls [70]. Martinez-Garcia et al. [71], for example, observed a clear association between obesity and fasting chemerin levels, but found no independent link between chemerin and PCOS itself. More recent studies also lack consistency. Bose et al. [72] demonstrated significantly higher chemerin levels in obese PCOS patients compared to lean PCOS patients but observed no difference between lean women with PCOS and lean controls [72]. Niepsuj et al. [70] likewise reported no significant differences between PCOS patients overall and controls; however, within the PCOS group, overweight and obese women had substantially higher chemerin levels than women of normal weight. They also noted positive correlations between chemerin and BMI, WHR, triglycerides, and fasting glucose, as well as a negative correlation with HDL-C [70]. In the study of Guvenc et al. [73], no significant differences in circulating chemerin between PCOS patients and the control group were found, but overweight women with PCOS had significantly higher levels of chemerin compared to women of a normal weight with PCOS. They also found no significant differences in chemerin levels between normal and overweight controls. It may, therefore, appear that BMI alone is not a predictive factor for serum chemerin levels. The results of a study conducted in a group of patients with hyperandrogenic and normoandrogenic PCOS phenotypes and a control group showed that chemerin levels were significantly higher in patients with the classic PCOS phenotype compared to patients with normoandrogenic PCOS and the control group. No differences in chemerin levels were observed between patients with the normoandrogenic PCOS phenotype and the control group. In this study, chemerin levels were positively correlated with testosterone levels, HOMA-IR, BMI, WHR, and the LH/FSH ratio [74].

Evidence on the relationship between serum chemerin and markers of IR remains mixed and partially contradictory. In our PCOS cohort, we observed a weak positive correlation between chemerin and glucose, and stronger correlations with HOMA-IR and insulin levels. Bose et al. [72] similarly found that serum chemerin concentrations were significantly elevated only in PCOS patients with type 2 diabetes or an impaired tolerance to glucose, while no differences were present between normoglycemic PCOS patients and controls. Despite the variability across studies, the overall literature suggests a meaningful relationship between chemerin and IR or dysglycemia in PCOS [12,70]. Chemerin may contribute to the development or worsening of IR, thereby promoting glucose metabolism disturbances and possibly type 2 diabetes in affected women.

Although a direct association between biochemical hyperandrogenism and circulating chemerin has not been firmly established, experimental evidence indicates that androgens can stimulate ovarian chemerin production, implying that chemerin may mediate some androgen-related effects on ovarian physiology and ovulatory function [11,65,75]. In our study, the PCOS group was normoandrogenic; nevertheless, we observed a weak positive correlation between chemerin and FAI, and a weak negative correlation with SHBG. An important aspect of our study is the observation that even in the absence of hyperandrogenism, women with PCOS phenotype D may exhibit increased inflammatory activity. This suggests the need to assess the prevalence of cardiovascular and metabolic risk factors in all PCOS phenotypes, not just in the classic forms. When they occur, it is advisable to implement appropriate therapeutic interventions such as lifestyle changes and, if needed, appropriate pharmacotherapy. It would also be advisable to consider the potential association between chemerin and thromboembolic diseases in PCOS by assessing the soluble fibrin monomer complex, which has not yet been evaluated to date and could provide an additional contribution to the understanding of this adipokine [76].

The limitations of this study include the small sample size and ethnic homogeneity. The homogeneous ethnicity of the study group, limited exclusively to the Polish population, restricts the generalizability of the results to other ethnically diverse populations. Further studies are needed in larger cohorts, considering various PCOS phenotypes and assessing chemerin concentrations, as well as other adipokines. The assessment should be performed both at diagnosis and after therapeutic interventions. This will clearly determine whether chemerin plays a role in the pathogenesis and course of PCOS or if it is simply associated with concomitant inflammation and metabolic disorders. Additionally, longitudinal studies are needed to more accurately assess the true epidemiological impact of abnormal chemerin levels on cardiometabolic outcomes.

## 5. Conclusions

Chemerin levels did not differ between women with phenotype D PCOS and age-, weight-, and BMI-matched controls. Chemerin correlated positively with metabolic alterations and hs-CRP and negatively correlated with SHBG in both groups. It positively correlated with FAI in the PCOS group; however, the correlations with FAI and SHBG were weak. These findings might suggest that chemerin levels are associated more with metabolic alterations than with hormonal status. Phenotype D PCOS represents a distinct end of the PCOS spectrum, differing substantially from classical phenotypes regarding metabolic abnormalities and adipokine profiles. The heterogeneity of PCOS—particularly variations in adiposity and hyperandrogenism across populations—may explain the inconsistent findings reported in the literature. Given the relatively small sample size, our results should be viewed as preliminary. Whether chemerin can be considered a reliable marker of metabolic status in PCOS remains an open question. Interventions targeting obesity may lead to favorable modulation of adipose tissue-derived adipokines, such as chemerin, which could be indicative of improved clinical outcomes. Overall, the adipokine profile may represent a valuable predictor of metabolic outcomes in patients with PCOS.

## Figures and Tables

**Table 1:** General characteristics of the women with polycystic ovary syndrome and the control group.

Group	PCOS (n = 49)			CG (n = 40)			*p*-Value
	Mean ± SD	Median	IQR	Mean ± SD	Median	IQR	
Age (years)	27.0 ±5.3	26.0	23.0–30.0	29.0 ±5.8	29.0	24.5–34.5	0.083
Weight (kg)	72.96 ± 21.4	66.0	58.0 ± 83.0	65.0 ± 15.9	59.5	55.0–69.0	0.065
BMI (kg/m^2^)	26.1 ± 7.6	23.6	20.7–29.8	27.7 ± 5.8	20.9	20.3–24.7	0.110
Waist circumference (cm)	84.4 ± 19.2	74.0	69.0–99.0	77.0 ± 15.4	70.0	69.0–81.0	0.034
Hip circumference (cm)	100.8 ± 15.2	97.0	90.0–110.0	94.4 ± 12.9	90.0	86.0–100.0	0.016
WHR	0.83 ± 0.1	0.80	0.76–0.92	0.81 ± 0.06	0.80	0.76–0.85	0.717
Adipose tissue (%)	34.5 ± 5.2	34.3	30.9–37.1	32.9 ± 5.6	31.3	28.9–34.4	0.086
Android fat (%)	31.2 ± 7.6	29.5	25.0–35.6	28.9 ± 7.9	27.5	22.3–32.8	0.198
Gynoid fat (%)	37.9 ± 3.9	37.9	34.8–40.5	36.4 ± 4.5	35.8	33.0–38.4	0.198 *

* t-Student test; PCOS—polycystic ovary syndrome; CG—control group; SD—standard deviations; IQR—interquartile range; BMI—body mass index; WHR—waist to hip ratio.

**Table 2:** Hormonal and biochemical profiles and chemerin concentration in women with polycystic ovary syndrome and the control group.

Group, Reference Ranges	PCOS (n = 49)			CG (n = 40)			*p*-Value
	Mean ± SD	Median	IQR	Mean ± SD	Median	IQR	
Chemerin (ng/mL)	216.5 ± 62.3	199.8	177.1–244.5	201.7 ± 45.5	205.5	172–224.9	0.480
Testosterone (ng/mL; 0.2–0.8)	0.37 ±0.1	0.36	0.3–0.4	0.29 ± 0.1	0.27	0.2–0.4	0.006
Androstendione (ng/mL; 0.3–3.5)	3.7 ± 1.4	3.9	2.7 ± 4.5	2.3 ± 0.9	2.3	1.9–2.7	<0.001
SHBG(nmol/L; 117–329)	51.2 ± 33.1	41.2	27.0–69.7	56.3 ± 22.2	54.45	36.2–69.1	0.089
FAI (0.55–12)	3.4 ± 2.4	2.5	1.6–4.4	2.2 ± 1.5	1.7	1.2–2.3	0.002
DHEA-S (µg/dL; 35–430)	300.9 ± 134.4	272.0	221.0–398.0	252.7 ± 113.8	240.5	174.5–336.5	0.081
LH (mIU/mL; 1.1–11.6)	8.7 ± 5.1	7.2	5.2–10.8	6.3 ± 3.1	5.6	4.3–8.7	0.038
FSH (mIU/mL; 2.8–11.6)	5.9 ± 2.1	5.8	4.5–7.3	6.4 ± 1.8	6.2	5.5 -7.1	0.251 *
hsCRP (mg/L; 0.2–5)	3.9 ± 8.7	1.0	0.3–3.0	1.4 ± 2.0	0.65	0.5–0.95	0.046
Total cholesterol (mg/dL; <190)	185.8 ± 37.1	178.5	156.5–207.5	180.0 ± 32.7	182.0	157.0–208.0	0.658
HDL cholesterol (mg/dL; >40)	63.8 ± 15.0	63.0	54.0–75.0	64.0 ± 15.4	63.0	56.0–69.0	0.842
LDL cholesterol (mg/dL; <135)	104.0 ± 34.0	98.0	81.0–118.5	102.6 ± 26.2	102.0	85.0–125.0	0.763
Triglycerides (mg/dL; <150)	88.0 ± 70.5	66.0	51.0–94.0	66.2 ± 33.0	57.0	46.0–73.0	0.127
Fasting glycemia (mg/dL; 70–99)	85.6 ± 7.5	85.6	80.0–92.0	86.0 ± 12.9	83.5	80.0–90.0	0.523
Insulin (µIU/mL; <24)	11.4 ± 16.7	7.1	3.8–10.7	6.8 ± 6.2	4.3	2.1–7.9	0.523
HOMA-IR	2.4 ± 3.5	1.5	0.8–2.5	1.4 ± 1.4	0.8	0.5–1.6	0.059

* t-Student test; PCOS—polycystic ovary syndrome; CG—control group; SD—standard deviations; IQR—interquartile range; BMI—body mass index; WHR—waist to hip ratio; SHBG—sex hormone binding globulin; FAI—free androgen index; DHEA-S—dehydroepiandrosterone sulfate; LH—luteinizing hormone; FSH—follicle-stimulating hormone; hsCRP—high sensitive C-reactive protein; HDL cholesterol—high-density lipoprotein cholesterol; LDL—low-density lipoprotein cholesterol; HOMA-IR—Homeostasis Model Assessment of Insulin Resistance.

**Table 3:** Correlations between chemerin concentration and biochemical, hormonal, and anthropometric parameters in the PCOS group.

PCOS	R	*p*
Age (years)	0.333	0.019
hsCRP (mg/L)	0.750	<0.001
Glucose (mg/dL)	0.313	0.029
Insulin (µIU/mL)	0.645	<0.001
HOMA-IR	0.665	<0.001
SHBG (nmol/L)	−0.388	0.006
FAI	0.375	0.009
Triglycerides (mg/dL)	0.600	<0.001
HDL cholesterol (mg/dL)	−0.418	0.003
Body weight (kg)	0.745	<0.001
BMI (kg/m^2^)	0.752	<0.001
Waist circumference (cm)	0.672	<0.001
%adipose tissue	0.704	<0.001
%android fat	0.603	<0.001

PCOS—polycystic ovary syndrome; hsCRP—high sensitive C-reactive protein; HOMA-IR—Homeostasis Model Assessment of Insulin Resistance; SHBG—sex hormone binding globulin; FAI—free androgen index; HDL cholesterol—high-density lipoprotein cholesterol; BMI—body mass index; WHR—waist to hip ratio; DHEA-S—dehydroepiandrosterone sulfate; LH—luteinizing hormone; FSH—follicle-stimulating hormone; LDL—low-density lipoprotein cholesterol; r—Pearson’s correlation coefficient.

**Table 4:** Correlations between chemerin concentration and biochemical, hormonal, and anthropometric parameters in the control group.

Control Group	R	*p*
hsCRP (mg/L)	0.552	<0.001
Insulin (μIU/mL)	0.411	0.010
SHBG (nmol/L)	−0.380	0.019
Triglycerides (mg/dL)	0.414	0.011
HDL cholesterol (mg/dL)	−0.382	0.020
BMI (kg/m^2^)	0.385	0.014
%adipose tissue	0.575	<0.001

hsCRP—high sensitive C-reactive protein; SHBG—sex hormone binding globulin; HDL cholesterol—high-density lipoprotein cholesterol; BMI—body mass index; r—Pearson’s correlation coefficient.

## Data Availability

The original contributions presented in the study are included in the article; further inquiries can be directed to the corresponding authors.
